# Anti-Vascular Endothelial Growth Factor Combined with Ocular Steroid Therapy for Persistent Diabetic Macular Edema: A Systematic Review and Meta-Analysis

**DOI:** 10.3390/ph17121574

**Published:** 2024-11-23

**Authors:** Yunxi Ma, Yunhan Tao, Mingzhu Yuan, Xufang Sun

**Affiliations:** Department of Ophthalmology, Tongji Hospital, Tongji Medical College, Huazhong University of Science and Technology, 1095 Jie-Fang Road, Wuhan 430030, China; karinema@foxmail.com (Y.M.); tyhsomeway@163.com (Y.T.); mzyuan0122@163.com (M.Y.)

**Keywords:** anti-vascular endothelial growth factor, combination therapy, meta-analysis, persistent diabetic macular edema, steroids

## Abstract

**Purpose**: Our purpose was to appraise the efficacy and safety of intravitreous vascular endothelial growth factor inhibitor (anti-VEGF) therapy combined with steroids for persistent diabetic macular edema. **Methods**: A systematic review was conducted of the research evaluating the combination therapy of anti-VEGF and steroids for persistent diabetic macular edema compared to anti-VEGF alone. A meta-analysis was performed using a protocol registered in PROSPERO (CRD42023476333). Continuous and binary variables were extracted. Results were expressed as the mean difference (MD) and risk ratio (RR). **Results**: A total of 9 trials with 537 eyes were included. The MDs of improvement in best-corrected visual acuity (BCVA) at 1/2/3/6/9/12 months between the combined and monotherapy groups were 1.33 (95% CI [−1.31,3.96]), 3.03 (95% CI [0.01, 6.06]), −0.37 (95% CI [−4.74, 4.00]), −1.37 (95% CI [−4.65, 1.91]), 1.05 (95% CI [−3.68, 5.77]), and 1.70 (95% CI [−3.52, 6.93]). The MDs concerned with a central retinal thickness (CMT) decline in at 1/2/3/6/9/12 months between the two groups were −47.33, 95% CI [−94.35, −0.32]), −89.19 (95% CI [−114.38, −64.00]), −58.84 (95% CI [−96.93, −20.74]), −57.23 (95% CI [−102.62, −11.84]), −40.59 (95% CI [−80.59, −0.58]), and −38.89 (95% CI [−77.38, −0.40]), respectively. Furthermore, the combined group obtained higher relative risks of experiencing events with high intraocular pressure and progressed cataracts. **Conclusions**: Anti-VEGF combined with ocular steroids showed a significant advantage in improving the retinal anatomical structure compared to anti-VEGF monotherapy for persistent diabetic macular edema. However, as the treatment period extended, the combination treatment was no more effective than monotherapy after 2 months, with more severe side effects.

## 1. Introduction

Diabetic macular edema (DME) accounts for significant visual impairment among the global workforce, with a global prevalence rate of 5.47% [1,2]. It affects vision due to abnormal metabolism, vascular leakage, and inflammation in the macular region [2].

Intravitreous anti-vascular endothelial growth factor (anti-VEGF), as the frontline therapeutic intervention for ME, improves vision and retinal anatomy [3]. Nevertheless, up to 65% of lesions presented with edema persisted during monthly injections over one year, often resulting in reduced visual acuity [4]. Continuous therapeutic injections are necessary for managing recurrent edema to preserve vision and regulate retinal exudation as the disease advances, leading to increased healthcare costs and risks of adverse effects [5,6,7]. Therefore, an additional ocular treatment is needed with a suboptimal response to anti-VEGF therapy.

Steroids, first used for ocular diseases in the 1950s, reduce inflammation and inhibit VEGF through various mechanisms [8,9]. Risks of posterior subcapsular cataracts and steroid-induced glaucoma have limited the widespread usage of steroids, and most physicians consider intraocular corticosteroid regimens as an alternative therapy [10,11]. In the OASIS and BEVORDEX trials, the dexamethasone implant (DEX) demonstrated superior outcomes and a higher incidence of steroid-induced hypertension compared to bevacizumab [12,13]. Switching to DEX was also suggested to achieve short-term anatomical and functional improvement for refractory DME [14].

Combination therapies of anti-VEGF and steroids target various pathophysiological mechanisms and may be an attractive alternative for persistent DME. Meanwhile, combination therapy aims to overcome shortcomings simultaneously, providing a stronger influence and long-lasting efficacy. In the DexaBe study, improvements in central retinal thickness were found, while vision remained stable or improved with the combination treatment [15]. A common consensus on combination therapy for persistent DME treatments has not yet been reached. This project aims to explore visual and anatomic functions of anti-VEGF monotherapy compared to anti-VEGF combined with steroids for DME therapy. Further analyses were conducted to appraise the effects of different etiologies and retreatment frequencies, aiming to provide clinical references.

## 2. Methods

### 2.1. Study Design and Search Strategy

This study was conducted strictly following the guidelines of Systematic Reviews and Meta-Analyses (PRISMA) and was registered in PROSPERO (CRD42023476333). Candidate articles and clinical trials were screened in databases including PubMed, Embase, Cochrane Library, and Web of Science from the inception to 26 February 2024. We searched for the following keywords and free text terms using MESH: Anti-Vascular Endothelial Growth Factors, Adrenal Cortex Hormones, Macular Edema, and Intravitreal. No limitations were imposed on searching for studies. Detailed search strategies arere referenced in Supplementary File S1.

### 2.2. Inclusion and Exclusion Criteria

Eligible studies possessed the following criteria: 1. Patients over 18 years old with diagnosed persistent diabetic macular edema are defined by a lack of discernible enhancement in best-corrected visual acuity (BCVA) or a decrease in central macular thickness (CMT) of less than 10% after a minimum of three anti-VEGF injections. 2. CMT is defined as the thickness from the retinal fovea to the retinal pigment epithelium (RPE), measured by optical coherence tomography (OCT) following The Diabetic Retinopathy Clinical Research Network. 3. Comparisons between monotherapy of anti-VEGF and a combination of anti-VEGFs and steroids. 4. Eyes without ocular pharmacotherapy or laser photocoagulation of the retina within the previous 3 months. 5. Retrospective and non-randomized controlled trials (non-RCTs) were eligible when the number of RCTs was limited. 6. Trials reported over one of the following main results: (1) BCVA changes, (2) CMT thickening, (3) elevated intraocular pressure (IOP), and (4) aggravated cataracts.

The exclusion criteria are as follows: 1. History of glaucoma, eye injuries, vitrectomy, severe systemic diseases, and systemic medication use of anti-VEGFs or steroids. 2. Duplications, reviews, case reports, letters, and non-English articles. 3. Studies with an insufficient sample size.

### 2.3. Data Extraction and Quality Assessment

The authors (YM, YT, and MY) completed full-text reviews and evaluated articles for eligibility independently. Any discrepancies were promptly rectified through discussion or were arbitrated by Prof. Sun. Data from the included studies were extracted or calculated in a consistent format, which contained the first author(s), publication year, country, research design (masking, stochastic approach), baseline parameters of patients in both groups (age, gender, etiology), interventions (groups, medications, injection schedules), sample size, and outcome measures (BCVA, CMT, high IOP, cataract progression) during follow-up periods (1/2/3/6/9/12 months). In this meta-analysis, BCVAs were recorded as The Early Treatment Diabetic Retinopathy Study (ETDRS) letters using a transformation formula that accounted for various forms of vision expression [16,17]. CMT was consistently referred to as central retinal thickness in the pooled articles discussing its various measurements. High IOP was consistently categorized as an event and contained in the analysis, akin to advancements in cataracts. The results of BCVA and CMT changes were expressed as means with standard deviation (SD).

Independent reviewers (YM, YT, and MY) employed various assessments for the risk of bias. Based on PRISMA recommendations, the quality of RCTs was evaluated by the Cochrane Collaboration tool 2.0 [18]. The Methodological Index for Non-Randomized Studies (MINORS) was applied to evaluate non-RCTs and retrospective studies [19].

### 2.4. Heterogeneity and Sensitivity Analysis

The primary variable that differed among the studies was pharmacotherapy. Therefore, studies were grouped according to the frequency of anti-VEGF therapy at each follow-up visit following the initial pooled analyses. The subgroup results were presented in the primary meta-analysis. To further investigate potential contributors to heterogeneity, subgroup analyses were applied under different anti-VEGFs (IVA, IVB, IVR) and study types (RCT, non-RCT, retrospective trials). The one-by-one elimination method was performed for sensitivity analysis on overall trials at each study point, but it was not conducted for small sample sizes of subgroups. We performed Egger’s test and Begg’s test for publication bias involving continuous variables [20,21]. For binary variables, Harbord’s test was added [22].

### 2.5. Statistical Analysis

For continuous outcomes including BCVA and CMT, mean differences (MDs) with a 95% confidence interval (CI) were calculated. For binary variables involving elevated IOP and progressed cataract events, the risk ratio (RR) with a 95% CI was calculated. An analysis of the subgroups according to the frequency of anti-VEGF therapy at follow-up visits was conducted, including pairwise comparisons. The *p* values of Cochran’s Q-test and I^2^ test were both calculated for heterogeneity analysis according to the standard chi-square test. The fixed-effect model was applied when statistics showed homogeneity (I^2^ < 50%, *p* > 0.05) [23]. Otherwise, the random-effects model would be used [24]. Statistical significance was defined as *p* < 0.05. The Stata/SE software release 15.1 (Stata/SE, College Station, TX, USA) was applied for all statistical analyses.

## 3. Results

### 3.1. Study Characteristics

**Figure 1:**
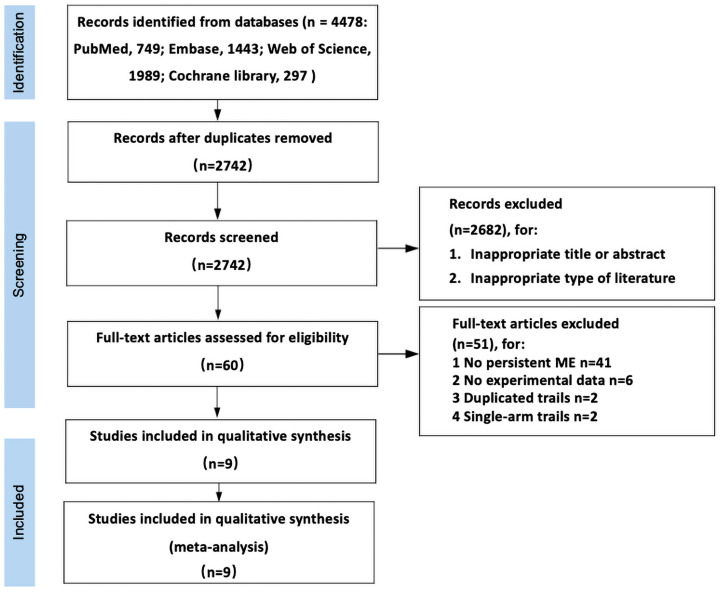
Flowchart depicting the search process for eligible studies.

The initial retrieval obtained 4478 records in total, of which 1736 duplicated references were eliminated. Sixty records were obtained by screening detailed titles and abstracts. Eventually, nine full-text articles were identified for the current meta-analysis. These articles consisted of four dual-arm RCTs, one three-arm RCT, two dual-arm non-RCTs, and two dual-arm retrospective trials, as shown in Figure 1 [25,26,27,28,29,30,31,32,33,34]. In total, 537 eyes were retained in this meta-analysis. The primary measurements (BCVA/CMT/adverse events) were recorded during follow-up visits ranging from 3 to 12 months. Detailed information on all trials is summarized in Table 1.

### 3.2. Best-Corrected Visual Acuity

#### 3.2.1. One Month

**Figure 2:**
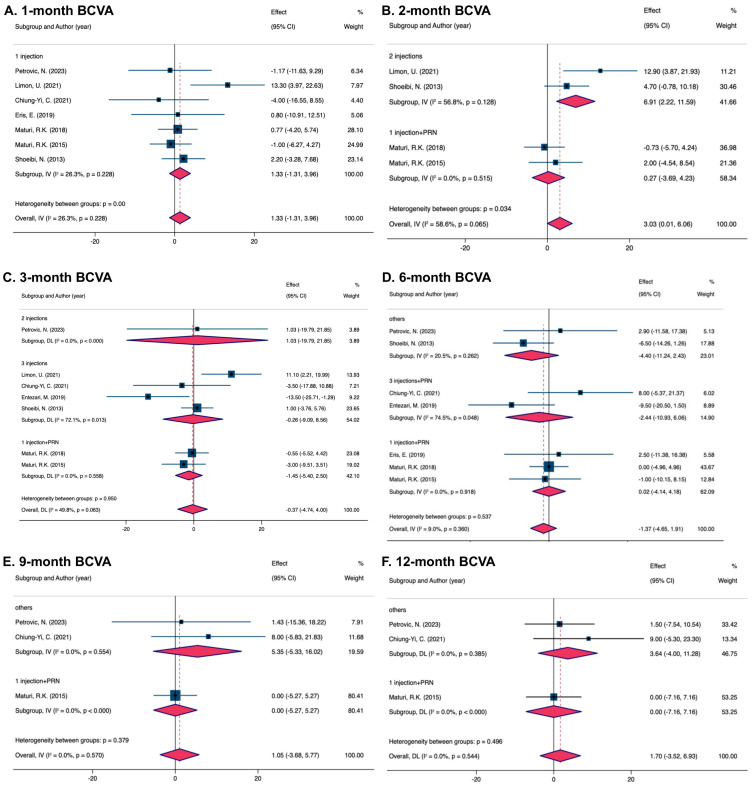
Forest plots showing effectiveness of combined treatment versus monotherapy on BCVA at various intervals [25,27,28,29,30,31,33,34]. (**A**) 1 month; (**B**) 2 months; (**C**) 3 months; (**D**) 6 months; (**E**) 9 months; (**F**) 12 months.

Pooling results from seven studies showed no significant difference in BCVA in both the combined and monotherapy groups at the 1-month visit (MD = 1.33, 95% CI [−1.31, 3.96]). As all patients included received a single anti-VEGF injection, regardless of the various medication protocols used, the group was uniformly categorized as having received “1 injection”. No detection of heterogeneity was detected for the 1-month therapy (I^2^ = 26.3%) (Figure 2A).

#### 3.2.2. Two Months

The results pooled from four trials demonstrated a better improvement in BCVA within the combined group after the 2-month treatment (MD = 3.03, 95% CI [0.01, 6.06]). Heterogeneity in pooling data was detected (I^2^ = 58.6%). Subgroups were defined according to the frequency of injections received during the 2-month follow-up as “2 injections” and “1 injection + PRN”. The “2 injections” subgroup (MD = 6.91, 95% CI [2.22, 11.59]) matched the overall results but indicated a larger MD. Although the MD of the “1 injection + PRN” subgroup was much smaller compared to the total MD, no significant difference existed (MD = 0.27, 95% CI [−3.69, 4.32]). Heterogeneity was detected in the “2 injections” subgroup (I^2^ = 56.8%) but not in the “1 injection + PRN” subgroup (I^2^ = 0%) (Figure 2B).

#### 3.2.3. Three Months

The pooled results from seven studies showed no significant differences in BCVA between the combined and mono-treated groups after 3 months (MD = −0.37, 95% CI [−4.74, 4.00]). No heterogeneity existed (I^2^ = 49.8%). The research was divided into different subgroups based on various injection schedules of anti-VEGFs, including “2 injections”, “3 injections”, and “1 injection + PRN”. Similarities were found in the MD of the subgroups “3 injections” (MD = −0.26, 95% CI [−9.09, 8.56]) and “1 injection + PRN” (MD = −1.45, 95% CI [−5.40, 2.50]), although statistical differences were significant (Figure 2C).

#### 3.2.4. Six Months

The pooled analysis of seven trials showed an insignificant difference in BCVA between two groups (MD = −1.37, 95% CI [−4.65, 1.91]), and no heterogeneity was observed (I^2^ = 9.0%). The subgroups were labeled according to the numbers of anti-VEGF therapy as “Others”, “3 injections + PRN”, and “1 injection + PRN”. “Others” were identified as different injection frequencies. Outcomes from the subgroups “1 injection + PRN” (MD = 0.02, 95% CI [−4.14, 4.18]), “3 injections + PRN” (MD = −2.44, 95% CI [−10.93, 6.06]), and “Others” (MD = −4.40, 95% CI [−11.24, 2.43]) all showed no significant difference in BCVA improvement (Figure 2D).

#### 3.2.5. Nine Months

The overall MD from three pooled studies after 9 months was 1.05 (95% CI [−3.68, 5.77]). Heterogeneity was not detected (I^2^ = 0%). The research was divided into different subgroups under the anti-VEGF injection frequencies after 9 months, categorized as “1 injection + PRN” and “Others”. Outcomes of both subgroups showed no significant difference in BCVA progress (“1 injection + PRN”: MD = 0.00, 95% CI [−5.27, 5.27]; “Others”: MD = 5.35, 95%CI [−5.33, 16.02]) (Figure 2E).

#### 3.2.6. Twelve Months

Three trials were combined during the 12-month visits. The whole MD was 1.70 (95% CI [−3.52, 6.93]). The analyses were homogeneous (I^2^ = 0%). Studies were divided into the subgroups “1 injection + PRN” and “Others”. Differences in BCVA between the subgroup “1 injection + PRN” (MD = 0.00, 95% CI [−7.16, 7.16]) and “Others” (MD = 3.64, 95% CI [−4.00, 11.28]) showed no significant difference in BCVA progress (Figure 2F).

### 3.3. Central Retinal Thickness

#### 3.3.1. One Month

**Figure 3:**
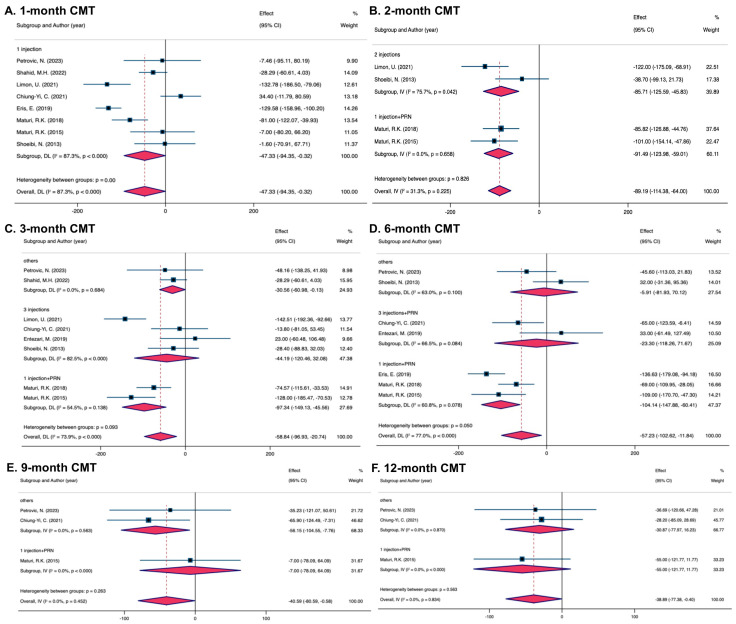
Forest plots showing the effectiveness of combined treatment versus monotherapy on CMT at various intervals [25,26,27,28,29,30,31,33,34]. (**A**) 1 month; (**B**) 2 months; (**C**) 3 months; (**D**) 6 months; (**E**) 9 months; (**F**) 12 months.

The overall outcomes of eight trials showed a significant decline in CMT within the combination group after the 1-month visits (MD = −47.33, 95% CI [−94.35, −0.32]). Overall trials were uniformly labeled as “1 injection”. Heterogeneity was detected for the 1-month visits (I^2^ = 87.3%) (Figure 3A).

#### 3.3.2. Two Months

Outcomes pooled from four trials demonstrated a greater decline in CMT within the combined group receiving the 2-month treatment (MD = −89.19, 95% CI [−114.38, −64.00]). Heterogeneity of pooled data was not found (I^2^ = 31.3%). Subgroups were defined according to the frequency of anti-VEGF therapy sessions after the 2-month visits as “2 injections” and “1 injection + PRN”. The “1 injection + PRN” subgroup (MD = −91.49, 95% CI [−123.98, −59.01]) matched the total outcomes, while the MD in the subgroup “2 injections” (MD = −85.17, 95% CI [−125.59, −45.83]) was greater than the total MD. All statistical differences were significant (Figure 3B).

#### 3.3.3. Three Months

After 3 months, the combined therapy group’s CMT regression was better in the total results of eight studies (MD = −58.84, 95% CI [−96.93, −20.74]). There was complete heterogeneity (I^2^ = 73.9%). Following the anti-VEGF injection numbers, the trials were split into three subgroups: “3 injections”, “1 injection + PRN”, and “Others”. Reductions were observed in the majority of the groups except the subgroup “3 injections” (MD = −44.19, 95% CI [−120.46, 32.08]). In comparison to the overall data, the subgroup “1 injection + PRN” showed a higher CMT decrease (MD = −97.34, 95% CI [−149.13, −45.56]), while the subgroup “Others” showed a lower MD of −30.55 (95%CI [−60.98, −0.13]). The subgroup “Others” (I^2^ = 0%) showed homogeneity, whereas “1 injection + PRN” (I^2^ = 54.5%) and “3 injections” (I^2^ = 82.5%) did not. An insignificant CMT decrease was showed in pairwise comparisons between “1 injection + PRN” and “3 injections” (Figure 3C).

#### 3.3.4. Six Months

A better CMT decrease was seen in the combined group (MD = −57.23, 95% CI [−102.62, −11.84]), while heterogeneity was found (I^2^ = 77.0%) upon the pooled results from seven trials. The subgroups were designated with labels such as “Others”, “3 injections + PRN” and “1 injection + PRN” based on the same guidelines. While the subgroups “3 injections + PRN” (MD = −23.30, 95% CI [−118.26, 71.67]) and “Others” (MD = −5.91, 95% CI [−81.93, 70.12]) showed an insignificant distinction, the outcomes of the subgroup “1 injection + PRN” (MD = −104.14, 95% CI [−147.88, −60.41]) suggested larger declines in CMT than the total study. The subgroups “1 injection + PRN” (I^2^ = 60.8%), “3 injections + PRN” (I^2^ = 66.5%), and “Others” (I^2^ = 63.0%) showed heterogeneity. An insignificant CMT decease was seen in pairwise comparisons between “1 injection + PRN” and “3 injections + PRN” (Figure 3D).

#### 3.3.5. Nine Months

The overall MD from three pooled studies for CMT reduction at 9 months was −40.59 (95% CI [−80.59, −0.58]). Analyses were homogeneous (I^2^ = 0%). The trials were divided into the subgroups “Others” and “1 injection + PRN”. The “Others” subgroup (MD = −56.15, 95% CI [−104.55, −7.76]) showed a greater CMT reduction with combined therapy, whereas the “1 injection + PRN” subgroup (MD = −7.00, 95% CI [−78.09, 64.09]) showed no significance in CMT reduction. The number and total number of subgroups included limited pairwise comparisons (Figure 3E).

#### 3.3.6. Twelve Months

Three studies were pooled at 12-month visits. The overall MD was −38.89 (95%CI [−77.38, −0.40]). Analyses were homogeneous (I^2^ = 0%). The subgroups were designated with labels such as “Others” and “1 injection + PRN”. The subgroup “1 injection + PRN” (MD = −55.00, 95% CI [−121.77, 11.77]) showed a better CMT reduction in the combination group while insignificant. The results in the subgroup “Others” (MD = −24.04, 95% CI [−72.31, 24.22]) also showed no significance. The number and total number of subgroups included limited pairwise comparisons (Figure 3F).

### 3.4. Incidence of Adverse Events

**Figure 4:**
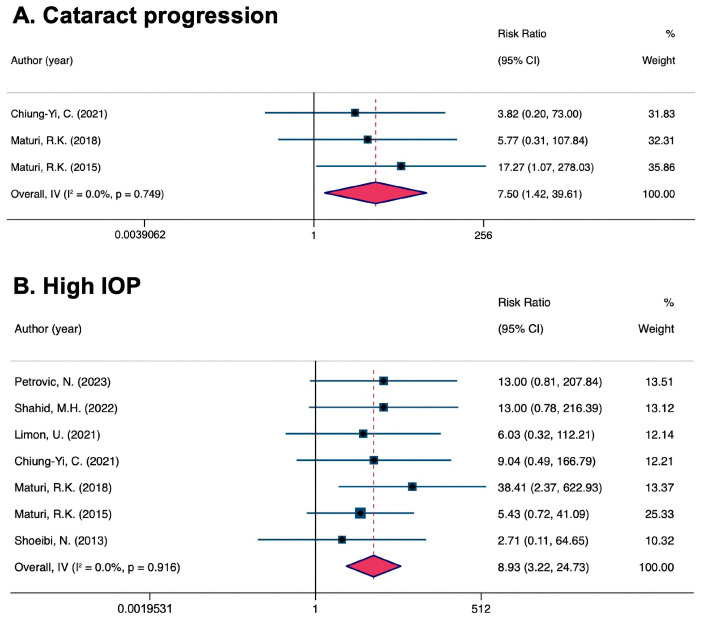
Comparison of the risk ratio of adverse events [25,26,27,28,31,33,34]. (**A**) Cataract progression; (**B**) high IOP.

Eligible studies reported the incidence of abnormal IOP elevation and worsening cataracts, which are common adverse events associated with intravitreal pharmacotherapy. Incidence rates were assessed for the safety of combined and monotherapy. Trials with no adverse events were excluded from the safety analysis to reduce the impact on cataract rates. Pseudophakic eyes were also excluded. Detailed adverse events in the overall trials are shown in the Supplementary File S2 (Tables S1–S3). The overall RR from three pooled studies (RR = 7.50, 95% CI [1.42, 39.61]) showed that combination therapy worsened cataracts more than monotherapy. There was no heterogeneity (I^2^ = 0%). The pooled results from seven studies showed a statistically significant increased risk of IOP elevation with combination therapy (RR = 8.93, 95% CI [3.22, 24.73]). The analyses were homogeneous (I^2^ = 0%) (Figure 4).

### 3.5. Heterogeneity Analysis

#### 3.5.1. Subgroup Analysis of Drug Types

BCVA in the anti-VEGF subgroup “IVR” at the 2-month visits showed an insignificant difference between the combination and monotherapy groups, which differed from the overall results with statistical significance. Results including CMT of “IVA” and “IVR” at the 1-month, 6-month, 9-month, and 12-month visits also changed to insignificant between the combination and monotherapy groups. For adverse events, statistical insignificance appeared in the “IVA” subgroup of high IOP events and the “IVR” subgroup of cataract progression. The remaining groups matched the overall results. The results indicated that the drug type was not a source of heterogeneity (Supplementary File S2: Figures S1, S2, and S5).

#### 3.5.2. Subgroup Analysis by Study Type

The pooled BCVA results from RCTs were not significant at the 2-month visits, whereas results from non-RCTs were significant compared with the overall results. CMT analyses were statistically insignificant in non-RCTs and retrospective studies at 1-month visits, in retrospective studies at 3-month visits, and in RCTs and non-RCTs studies at 6-month and 9-month visits. The remaining results matched the overall analysis. The results showed that the trial type did not contribute to heterogeneity (Supplementary File S2 Figures S3–S5).

### 3.6. Quality and Risk-of-Bias Assessment

Summaries of the risk-of-bias assessments are provided in the Supplement. The quality of 5 included RCTs [26,30,31,32,33,34] was evaluated through the Cochrane Collaboration tool 2.0 [18]. Deviations from the intended interventions were not reported in 2 RCTs, concerns about missing outcome data were raised in 1 RCT, uncertainties about outcome measurement methods were raised in 5 RCTs, and concerns about the selection of reported outcomes were raised in 1 RCT.Therefore, the overall bias assessment was rated as “Some concerns”, although the risk of bias was high in one study under certain conditions (Supplementary File S2: Figure S6). Non-RCTs and retrospective studies were assessed and graded using MINORS. One retrospective study received a score of 17 points, while the remaining studies scored >18, indicating an overall high quality (Supplementary File S2: Table S4).

### 3.7. Sensitivity Analysis and Publication Bias

**Figure 5:**
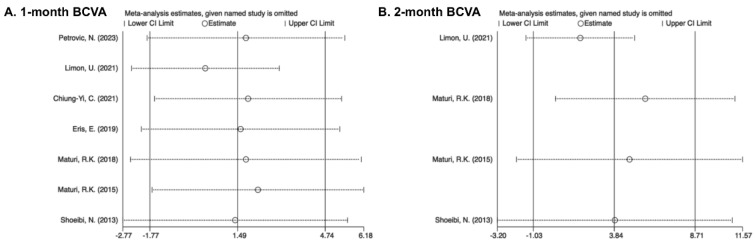

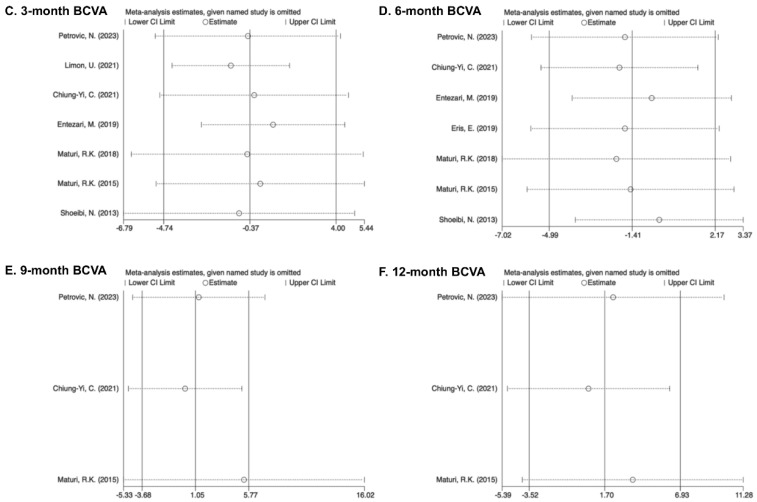
Sensitivity analysis of BCVA [25,27,28,29,30,31,33,34]. (**A**) 1 month; (**B**) 2 months; (**C**) 3 months; (**D**) 6 months; (**E**) 9 months; (**F**) 12 months.

**Figure 6:**
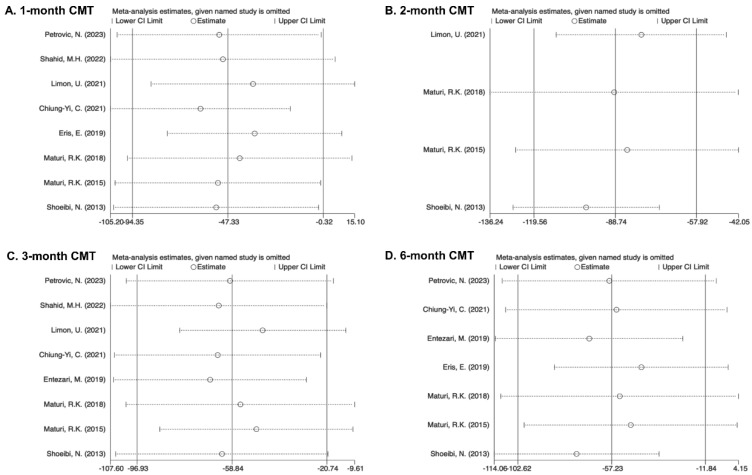

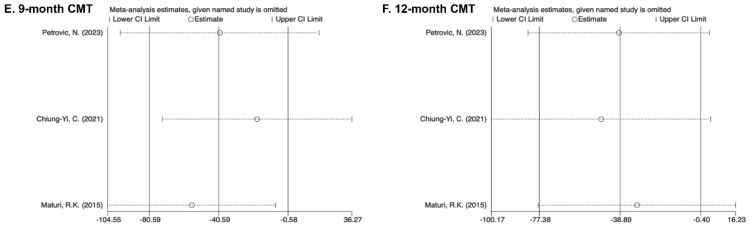
Sensitivity analysis of CMT [25,26,27,28,29,30,31,33,34]. (**A**) 1 month; (**B**) 2 months; (**C**) 3 months; (**D**) 6 months; (**E**) 9 months; (**F**) 12 months.

In sensitivity analyses, the overall results did not change significantly after excluding individual studies, indicating the stability of outcomes. Begg’s test, Egger’s test, and Harbord’s test were used for indicators such as BCVA, CMT, and adverse events. The results showed no evidence of publication bias (*p* > 0.1) (Figure 5 and Figure 6).

## 4. Discussion

Persistent diabetic macular edema, unresponsive to initial treatment with anti-VEGF agents, is attributed to its complex and heterogeneous inflammatory mechanisms [1]. Repeated intravitreal injections are insufficient to improve vision acuity and anatomical structure and are linked to increased economic burdens and risks of adverse events, including retinal detachment, vitreous hemorrhage, and endophthalmitis. In addition, retinal pigment epithelial damage and choroidal atrophy correlate with the frequency of anti-VEGF injections [35,36]. Ocular steroids have a broad anti-inflammatory spectrum with a long half-life [9,37]. Therefore, the inclusion of ocular steroids offers the possibility of reducing the number of injections, making it a valuable adjunct to therapy [38]. Studies have been controversial regarding the additional visual benefit of combination therapy and an increased risk of adverse events [31,39]. A meta-analysis comparing anti-VEGF plus steroids and anti-VEGF alone has not been performed.

This systematic review and meta-analysis aimed at evaluating treatments for persistent DME. The analysis of nine eligible clinical trials involving 537 eyes suggested that anti-VEGF combined with steroids was more effective than anti-VEGF alone. Further analyses were performed to investigate the specific efficacy of combination therapy and monotherapy during the follow-up period when therapy with monthly injections was required. To do this, the trials were divided into different groups based on the number of injections given at different intervals. The results showed that the combination treatment achieved better BCVA progression with statistical significance at 2-month intervals. As the “2 injections” subgroup achieved significantly greater MD than the “1 injection + PRN” subgroup at 2-month visits, monthly injections of anti-VEGF were recommended to increase the BCVA benefits of combined pharmacotherapy in the early treatment phase. Results of statistical insignificance in events were observed at each of the follow-up intervals except the 2-month point, identifying benefits in BCVA progression of combination medications at an early stage with a gradual decline in efficacy over time. For CMT, the combination regiments showed a consistent and significant reduction throughout all of the 12-month visits, which was distinct from BCVA. Recommendations for additional anti-VEGF injections were applicable for improvements in CMT. The results concluded that there were benefits to the retinal anatomical structure with combination therapy, while the efficacy of BCVA gradually decreased at long-term visits.

The results of the heterogeneity analysis showed that neither medications nor study design accounted for the heterogeneity that persisted within certain subgroups. The similarity of the BCVA and CMT changes between results classified as “IVA”, “IVB”, and “IVR” remained consistent with the overall results. The pharmaceutical factors did not reduce the heterogeneity observed between the pertinent subgroups. The results of the different study types differed for BCVA from 1 month to 6 months and for CMT at 2 months and 3 months. The inclusion of non-RCTs, retrospective studies, and the limited number of studies within each subgroup may affect the reliability of the results. Although RCTs were favored in terms of accuracy and applicability, the majority of the other research included met the MINORS quality criteria. Despite the variations observed in some groups, their results were consistent with pooled outcomes. Trial type and medication were not the source of heterogeneity, which may be due to factors inherent in the research differences in populations and protocols.

Sensitivity analysis for BCVA revealed the instability of the results across the 2-month follow-up points. The removal of “Maturi, R.K. (2018)” [31] results in consistent findings with the pooled results, thereby further highlighting that heterogeneity arises from variations among individual studies. The CMT results were found to be unstable at four time points, but their impact on the final pooled value was minimal, also reflecting a correlation with individual variations.

Currently, there is a paucity of long-term research in refractory macular edema, with limited evidence on the sustained efficacy and adverse events associated with anti-VEGF plus steroids compared to monotherapy. In a meta-analysis of DME, combination therapy showed little superiority over monotherapy in terms of long-term improvement in visual acuity [40]. In addition, steroid administration worsened cataract progression in more patients, leading to a deterioration in visual acuity during the follow-up period [31,34]. The meta-analysis showed that 50% of the studies exhibited a decrease in BCVA due to the cataract progression. In addition, studies showed the occurrence of steroid-induced ocular hypertension [30,31,32].

Intravitreal anti-VEGF injections carry the potential risk of increasing intraocular pressure [35,41]. A network meta-analysis within 26 RCTs found no statistically significant association between intravitreous anti-VEGF treatment and the occurrence of ocular hypertension, which is consistent with our results [42]. Our results showed a significantly increased relative risk of ocular hypertension and worsening cataract events with combination therapy, which was confirmed by a sensitivity analysis. Further treatments were recommended for adverse effects during the follow-up periods.

Limitations exist in this meta-analysis. Firstly, although the range of literature types was extended, the number of eligible trials with persistent DME was insufficient. Secondly, further subgroup classification based on detailed variables resulted in smaller group sample sizes, which compromised reliability. The inclusion of non-RCTs and retrospective studies also undermined the credibility. Future standardized RCTs of bigger sample sizes and multicenter plans are recommended for validation of our analysis. Thirdly, randomization methods and other research protocols were not adequately explained, and the inclusion criteria were not consistent. There were cases in which both eyes of a participant were included without efficacy adjustments, which affected the overall results. Fourthly, systematic errors from different trial devices in this meta-analysis limited genuine variation. A reduction in errors and standardization in measurements is essential in future studies. Fifthly, few studies have reported long-term pharmacotherapy effects, which reduces the credibility of the long-term treatment effects in this meta-analysis.

## 5. Conclusions

The early combination of anti-VEGF plus steroids with subsequent additions of anti-VEGF therapy is an effective approach to the treatment of refractory diabetic macular edema when visual acuity and visual structure decline. The development of innovative targeted drugs is needed to improve the efficacy of the treatment of this condition in the future.

## Figures and Tables

**Table 1:** Main characteristics of all included studies. C: combined therapy; M: monotherapy; NA: not available; IVA: intravitreous aflibercept; IVB: intravitreous bevacizumab; IVR: intravitreous ranibizumab; IVTA: intravitreous triamcinolone acetonide; DEX: dexamethasone sustained-release implant; CLS-TA: suprachoroidal injection of triamcinolone acetonide; PSTA: posterior sub-Tenon injection of triamcinolone acetonide.

Author; Ref.	Year	Location	Design	Intervention Model	Masking	Stochastic Approach	Etiology	Gender	Modules of Intervention	Sample Size	Outcome Reported	Follow-Up Duration (Month)
Petrovic [25]	2023	Serbia	Non-RCT	Dual-arm	NA	Non-randomized	Persistent DME	C: male/female 6/6 M: male/female 5/7	C: IVA 2 mg/0.05 mL plus IVTA 10 mg/0.1 mL M: IVA 1.25 mg/0.05 mL	C: 12 M: 12	BCVA, CMT, IOP, adverse reactions	12
Shahid [26]	2022	Pakistan	RCT	Dual-arm	Single-masked	Randomization	Persistent DME	Total: male/female 26/14	C: IVB 1.25 mg/0.05 mL plus CLS-TA 2 mg/0.05 mL M: IVB 1.25 mg/0.05 mL	C: 20 M: 20	CMT, adverse reactions	3
Limon, U. [27]	2021	Turkey	Non-RCT	Dual-arm	NA	Non-randomized	Persistent DME	C: male/female 12/17 M:male/female 14/16	C: IVB 1.25 mg/0.05 mL plus DEX 0.7 mg M: IVB 1.25 mg/0.05 mL	C: 35 M: 30	BCVA, CMT, IOP, adverse reactions	3
Chiu [28]	2021	China	Retrospective	Dual-arm	NA	NA	Persistent DME	C: male/female 10/13 M: male/female 14/6	C: IVR 0.5 mg plus PSTA 40 mg M: IVR 0.5 mg	C: 23 M: 20	BCVA, CMT, adverse reactions	12
Entezari [30]	2019	Iran	RCT	Dual-arm	Double-blind	Randomization	Persistent DME	C: male/female 8/11 M: male/female 5/10	C: IVB 1.25 mg/0.05 mL plus EPO 1000 μg/0.05 mL M: IVB 1.25 mg/0.05 ml	C: 24 M: 24	BCVA, CMT, adverse reactions	6
Eris [29]	2019	Turkey	Retrospective	Dual-arm	Open-label	Randomization	Persistent DME	NA	C: IVR 0.5 mg plus PSTA 40 mg M: IVR 0.5 mg	C: 38 M: 34	BCVA, CMT, IOP, adverse reactions	6
Maturi [31]	2018	America	RCT	Dual-arm	Double-masked	Randomization	Persistent DME	C: male/female 34/31 M: male/female 28/36	C: IVR 0.3 mg plus DEX 0.7 mg M: IVR 0.5 mg plus sham injection	C: 63 M: 64	BCVA, CMT, adverse reactions	6
Maturi [34]	2015	America	RCT	Dual-arm	Single-masked	Randomization	Persistent DME	Total: male/female 13/17	C: IVB 1.25 mg/0.05 mL plus DEX 0.7 mg M: IVB 1.25 mg/0.05 mL	C: 21 M: 19	BCVA, CMT, adverse reactions	12
Shoeibi [33]	2013	Iran	RCT	Three-arm	Tri-blind	Randomization	Persistent DME	C: male/female 7/8 M: male/female 7/9	C: IVB 1.25 mg/0.05 mL plus IVTA 2 mg/0.05 mL M: IVB 1.25 mg/0.05 mL	C: 41 M: 37	BCVA, CMT, adverse reactions	6

## Data Availability

The datasets generated during and/or analyzed during the current study are available from the corresponding author upon reasonable request.

## Supplementary Materials

The following supporting information can be downloaded at https://www.mdpi.com/article/10.3390/ph17121574/s1, Supplement File S1: Search strategy. Supplement File S2: Tables S1–S5 and Figures S1–S6: Table S1: The summary of abnormal IOP elevation events; Table S2: The summary of cataract progression events in phakic and pseudophakic eyes; Table S3: The summary of cataract progression events for 3 included trials; Table S4: Quality assessment of included studies through MINORS; Table S5: Results of publication bias test; Figure S1: Subgroup analysis for anti-VEGFs; Figure S2: Subgroup analysis for anti-VEGFs; Figure S3: Subgroup analysis for study type; Figure S4: Subgroup analysis for study type; Figure S5: Subgroup analysis for adverse events; Figure S6: Assessment of the quality of randomized controlled trials according to Cochrane Risk of Bias Tool for Randomized Controlled Trials.

## References

[1] Zhang J., Zhang J., Zhang C., Zhang J., Gu L., Luo D., Qiu Q. (2022). Diabetic Macular Edema: Current Understanding, Molecular Mechanisms and Therapeutic Implications. Cells.

[2] Daruich A., Matet A., Moulin A., Kowalczuk L., Nicolas M., Sellam A., Rothschild P.-R., Omri S., Gélizé E., Jonet L. (2018). Mechanisms of macular edema: Beyond the surface. Prog. Retin. Eye Res..

[3] Fleckenstein M., Schmitz-Valckenberg S., Chakravarthy U. (2024). Age-Related Macular Degeneration: A Review. JAMA.

[4] Jaffe G.J., Ying G.-S., Toth C.A., Daniel E., Grunwald J.E., Martin D.F., Maguire M.G. (2019). Macular Morphology and Visual Acuity in Year Five of the Comparison of Age-related Macular Degeneration Treatments Trials. Ophthalmology.

[5] Marques A.P., Ramke J., Cairns J., Butt T., Zhang J.H., Jones I., Jovic M., Nandakumar A., Faal H., Taylor H. (2022). The economics of vision impairment and its leading causes: A systematic review. eClinicalMedicine.

[6] Durand M.L. (2013). Endophthalmitis. Clin. Microbiol. Infect..

[7] Klein K.S., Walsh M.K., Hassan T.S., Halperin L.S., Castellarin A.A., Roth D., Driscoll S., Prenner J.L. (2009). Endophthalmitis After Anti-VEGF Injections. Ophthalmology.

[8] Jampol L.M. (1985). Pharmacologic Therapy of Aphakic and Pseudophakic Cystoid Macular Edema. Ophthalmology.

[9] Schmidt-Erfurth U., Garcia-Arumi J., Bandello F., Berg K., Chakravarthy U., Gerendas B.S., Jonas J., Larsen M., Tadayoni R., Loewenstein A. (2017). Guidelines for the Management of Diabetic Macular Edema by the European Society of Retina Specialists (EURETINA). Ophthalmologica.

[10] Campochiaro P.A., Han Y.S., Mir T.A., Kherani S., Hafiz G., Krispel C., Liu T.Y.A., Wang J., Scott A.W., Zimmer-Galler I. (2017). Increased Frequency of Topical Steroids Provides Benefit in Patients With Recalcitrant Postsurgical Macular Edema. Am. J. Ophthalmol..

[11] Madjedi K., Pereira A., Ballios B.G., Arjmand P., Kertes P.J., Brent M., Yan P. (2022). Switching between anti-VEGF agents in the management of refractory diabetic macular edema: A systematic review. Surv. Ophthalmol..

[12] Meyer J., Fry C., Turner A., Razavi H. (2022). Intravitreal dexamethasone versus bevacizumab in Aboriginal and Torres Strait Islander patients with diabetic macular oedema: The OASIS study (a randomised control trial). Clin. Exp. Ophthalmol..

[13] Mehta H., Fraser-Bell S., Nguyen V., Lim L.L., Gillies M.C. (2018). Short-term vision gains at 12 weeks correlate with long-term vision gains at 2 years: Results from the BEVORDEX randomised clinical trial of bevacizumab versus dexamethasone implants for diabetic macular oedema. Br. J. Ophthalmol..

[14] Yuan Q., Liu Y., Xu H., Gao Y., Qin L., Gou Y., Tao M., Zhang M. (2022). Efficacy and safety of single-dose dexamethasone implantation for patients with persistent diabetic macular edema: A systematic review and meta-analysis. Graefe’s Arch. Clin. Exp. Ophthalmol..

[15] Veiga Reis F., Dalgalarrondo P., Da Silva Tavares Neto J.E., Wendeborn Rodrigues M., Scott I.U., Jorge R. (2023). Combined intravitreal dexamethasone and bevacizumab injection for the treatment of persistent diabetic macular edema (DexaBe study): A phase I clinical study. Int. J. Retin. Vitr..

[16] Ferris F.L., Kassoff A., Bresnick G.H., Bailey I. (1982). New Visual Acuity Charts for Clinical Research. Am. J. Ophthalmol..

[17] Khoshnood B., Mesbah M., Jeanbat V., Lafuma A., Berdeaux G. (2010). Transforming scales of measurement of visual acuity at the group level: Visual acuity transformation. Ophthalmic Physiol. Opt..

[18] Sterne J.A.C., Savović J., Page M.J., Elbers R.G., Blencowe N.S., Boutron I., Cates C.J., Cheng H.-Y., Corbett M.S., Eldridge S.M. (2019). RoB 2: A revised tool for assessing risk of bias in randomised trials. BMJ.

[19] Slim K., Nini E., Forestier D., Kwiatkowski F., Panis Y., Chipponi J. (2003). Methodological index for non-randomized studies (*MINORS*): Development and validation of a new instrument. ANZ J. Surg..

[20] Egger M., Smith G.D., Schneider M., Minder C. (1997). Bias in meta-analysis detected by a simple, graphical test. BMJ.

[21] Begg C.B., Mazumdar M. (1994). Operating characteristics of a rank correlation test for publication bias. Biometrics.

[22] Harbord R.M., Egger M., Sterne J.A.C. (2006). A modified test for small-study effects in meta-analyses of controlled trials with binary endpoints. Stat. Med..

[23] Mantel N., Haenszel W. (1959). Statistical aspects of the analysis of data from retrospective studies of disease. J. Natl. Cancer Inst..

[24] DerSimonian R., Laird N. (1986). Meta-analysis in clinical trials. Control. Clin. Trials.

[25] Petrovic N., Todorovic D., Sarenac Vulovic T., Sreckovic S., Zivic F., Risimic D. (2023). Combined Treatment of Persistent Diabetic Macular Edema with Aflibercept and Triamcinolone Acetonide in Pseudophakic Eyes. Medicina.

[26] Shahid M.H., Rashid F., Tauqeer S., Ali R., Farooq M.T., Aleem N. (2022). Comparison of Suprachoroidal Triamcinolone Injection with Intravitreal Bevacizumab Vs Intravitreal Bevacizumab only in Treatment of Refractory Diabetic Macular Edema. Pak. J. Med. Health Sci..

[27] Limon U. (2021). Early effect of simultaneous intravitreal dexamethasone and bevacizumab combination treatment in patients with persistent diabetic macular edema. J. Fr. Ophtalmol..

[28] Chiu C.-Y., Huang T.-L., Chang P.-Y., Chen F.-T., Hsu Y.-R., Chen Y.-J., Wang J.-K. (2021). Combined intravitreal ranibizumab and posterior subtenon triamcinolone acetonide injections for patients with diabetic macular edema refractory to intravitreal ranibizumab monotherapy. Taiwan J. Ophthalmol..

[29] Eriş E., Perente I., Vural E., Vural A., Seymen Z., Celebi A.R.C., Erdogan G., Ozkaya A., Artunay O. (2019). Evaluation of the effect of combined intravitreal ranibizumab injection and sub-tenon steroid injection in the treatment of resistant diabetic macular edema. Int. Ophthalmol..

[30] Entezari M., Flavarjani Z.K., Ramezani A., Nikkhah H., Karimi S., Moghadam H.F., Daftarian N., Yaseri M. (2019). Combination of intravitreal bevacizumab and erythropoietin versus intravitreal bevacizumab alone for refractory diabetic macular edema: A randomized double-blind clinical trial. Graefe’s Arch. Clin. Exp. Ophthalmol..

[31] Maturi R.K., Glassman A.R., Liu D., Beck R.W., Bhavsar A.R., Bressler N.M., Jampol L.M., Melia M., Punjabi O.S., Salehi-Had H. (2018). Effect of Adding Dexamethasone to Continued Ranibizumab Treatment in Patients With Persistent Diabetic Macular Edema: A DRCR Network Phase 2 Randomized Clinical Trial. JAMA Ophthalmol..

[32] Rezar-Dreindl S., Eibenberger K., Buehl W., Georgopoulos M., Weigert G., Krall C., Dunavoelgyi R., Schmidt-Erfurth U., Sacu S. (2017). Role of Additional Dexamethasone for the Management of Persistent or Recurrent Neovascular Age-Related Macular Degeneration Under Ranibizumab Treatment. Retina.

[33] Shoeibi N., Ahmadieh H., Entezari M., Yaseri M. (2013). Intravitreal Bevacizumab with or without Triamcinolone for Refractory Diabetic Macular Edema: Long-term Results of a Clinical Trial. J. Ophthalmic Vis. Res..

[34] MATURI R.K., Bleau L., Saunders J., Mubasher M., Stewart M.W. (2015). A 12-Month, Single-Masked, Randomized Controlled Study of Eyes with Persistent Diabetic Macular Edema after Multiple Anti-Vegf Injections to Assess the Efficacy of the Dexamethasone-Delayed Delivery System as an Adjunct to Bevacizumab Compared with Continued Bevacizumab Monotherapy. Retina.

[35] Zehden J.A., Mortensen X.M., Reddy A., Zhang A.Y. (2022). Systemic and Ocular Adverse Events with Intravitreal Anti-VEGF Therapy Used in the Treatment of Diabetic Retinopathy: A Review. Curr. Diabetes Rep..

[36] Wells J.A., Glassman A.R., Ayala A.R., Jampol L.M., Bressler N.M., Bressler S.B., Brucker A.J., Ferris F.L., Hampton G.R., Jhaveri C. (2016). Aflibercept, Bevacizumab, or Ranibizumab for Diabetic Macular Edema. Ophthalmology.

[37] Chauhan M.Z., Rather P.A., Samarah S.M., Elhusseiny A.M., Sallam A.B. (2022). Current and Novel Therapeutic Approaches for Treatment of Diabetic Macular Edema. Cells.

[38] Cai X., Zhao J., Dang Y. (2024). Combination Therapy with Anti-VEGF and Intravitreal Dexamethasone Implant for Macular Edema Secondary to Retinal Vein Occlusion. Curr. Eye Res..

[39] Abdel-Maboud M., Menshawy E., Bahbah E.I., Outani O., Menshawy A. (2021). Intravitreal bevacizumab versus intravitreal triamcinolone for diabetic macular edema–Systematic review, meta-analysis and meta-regression. PLoS ONE.

[40] Mehta H., Hennings C., Gillies M.C., Nguyen V., Campain A., Fraser-Bell S. (2018). Anti-vascular endothelial growth factor combined with intravitreal steroids for diabetic macular oedema. Cochrane Database Syst. Rev..

[41] Ghasemi Falavarjani K., Nguyen Q.D. (2013). Adverse events and complications associated with intravitreal injection of anti-VEGF agents: A review of literature. Eye.

[42] Nanji K., Sarohia G.S., Kennedy K., Ceyhan T., McKechnie T., Phillips M., Devji T., Thabane L., Kaiser P., Sarraf D. (2022). The 12- and 24-Month Effects of Intravitreal Ranibizumab, Aflibercept, and Bevacizumab on Intraocular Pressure. Ophthalmology.

