# Supplementary material for: Anti-Vascular Endothelial Growth Factor Combined with Ocular Steroid Therapy for Persistent Diabetic Macular Edema: A Systematic Review and Meta-Analysis
Source: Pharmaceuticals (Basel). 2024 Nov 23;17(12):1574. doi: 10.3390/ph17121574 (PMC11679650; doi:10.3390/ph17121574)
Supplement: Supplementary file 1 [file pharmaceuticals-17-01574-s001.zip › pharmaceuticals-3270362-supplementary.pdf]

Supplement File S1—Search strategy.

Detailed search strategies:

For Pubmed：

((((("Vascular Endothelial Growth Factors"[Mesh]) OR (Anti-Vascular Endothelial Growth Factors)) OR (((((((((((((((((((((((((((((((((((((pegaptanib sodium) OR (Macugen)) OR (Ranibizumab)) OR (Lucentis)) OR (RhuFab V2)) OR (V2, RhuFab)) OR (Bevacizumab)) OR (Mvasi)) OR (Bevacizumab-awwb)) OR (Bevacizumab awwb)) OR (Avastin)) OR (Aflibercept)) OR (VEGF Trap - regeneron)) OR (VEGF Trap-Eye)) OR (VEGF-Trap)) OR (eylea)) OR (Zaltrap)) OR (AVE 0005)) OR (AVE0005)) OR (AVE-0005)) OR (AVE 005)) OR (AVE005)) OR (AVE-005)) OR (ZIV-aflibercept)) OR (Brolucizumab)) OR (Beovu)) OR (RTH258)) OR (RTH-258)) OR (ESBA1008)) OR (ESBA-1008)) OR (Susvimo)) OR (Abicipar pegol)) OR (anti-VEGF DARPin)) OR (Faricimab)) OR (Vabysmo)) OR (KH902 fusion protein)) OR (conbercept))) AND (("Adrenal Cortex Hormones"[Mesh]) OR (((Hormones, Adrenal Cortex) OR (Corticosteroids)) OR (Corticosteroid)) OR (Corticoids)) OR (Corticoids)) OR (Adrenal Cortex Hormone)) OR (Cortex Hormone, Adrenal)) OR (Cortex Hormone, Adrenal)) OR (Triamcinolone acetonide)) OR (Acetonide, Triamcinolone)) OR (Cinonide)) OR (Tricort-40)) OR (Tricort 40)) OR (Tricort40)) OR (Tricort40)) OR (Kenalog 40)) OR (Azmacort)) OR (Kenacort A)) OR (Triesence)) OR (dexamethasone intravitreal implant)) OR (dexamethasone implant)) OR (Ozurdex)) OR (Methylfluorprednisolone)) OR (Hexadecadrol)) OR (Decameth)) OR (Decaspray)) OR (Dexasone)) OR (Dexpak)) OR (Maxidex)) OR (Millicorten)) OR (Oradexon)) OR (Decaject)) OR (Decaject-L.A.)) OR (Decaject L.A.)) OR (Hexadrol)) OR (fluocinolone acetonide implant)) OR (Iluvien)) OR (Acetonide, Fluocinolone)) OR (Acetonide, Fluocinolone)) OR (Acetonide, Fluortriamcinolone)) OR (Flurosyn)) OR (Jellin)) OR (Jellisoft)) OR (Synalar)) OR (Synalar-HP)) OR (Synalar HP)) OR (Synemol)) OR (Synamol)) OR (Alvadermo)) OR (Capex)) OR (Co-Fluocin)) OR (Co Fluocin)) OR (Cortiespec)) OR (Derma-Smooth FS)) OR (Derma Smooth FS)) OR (Flucinar)) OR (Fluocid)) OR (Fluodermo)) OR (Fluonid)) OR (Fluotrex)) OR (Flusolgen)) OR (Gelidina)) OR (triamcinolone acetonide (CLS-TA))))) AND (("Macular Edema"[Mesh]) OR (((((((((((Edema, Macular) OR (Irvine-Gass Syndrome)) OR (Irvine Gass Syndrome)) OR (Syndrome, Irvine-Gass)) OR (Cystoid Macular Edema, Postoperative)) OR (Macular Edema, Cystoid)) OR (Edema, Cystoid Macular)) OR (Cystoid Macular Dystrophy)) OR (Macular Dystrophy, Dominant Cystoid)) OR (Central Retinal Edema, Cystoid)) OR (Cystoid Macular Edema)))) AND (("Intravitreal Injections"[Mesh]) OR (((Injection, Intravitreal) OR (Injections, Intravitreal)) OR (Intravitreal Injection)))

For Embase：

1 (Vascular Endothelial Growth Factors or VEGFs or Anti-Vascular Endothelial Growth Factors or Anti-VEGFs or pegaptanib sodium or Macugen or Ranibizumab or Lucentis or RhuFab V2 or V2, RhuFab or Bevacizumab or Mvasi or Bevacizumab-awwb or Bevacizumab awwb or Avastin or Aflibercept or VEGF Trap - regeneron or VEGF Trap-Eye or VEGF-Trap or eylea or Zaltrap or "AVE 0005" or AVE0005 or AVE-0005 or "AVE 005" or AVE005 or AVE-005 or ZIV-aflibercept or Brolucizumab or Beovu or RTH258 or RTH-258 or ESBA1008 or ESBA-1008 or Susvimo or Genentech or Abicipar pegol or anti-VEGF DARPin or Faricimab or Vabysmo or Tarcocimab or

KH902 fusion protein or conbercept).af.
2 (Adrenal Cortex Hormones or Hormones, Adrenal Cortex or Corticosteroids or Corticosteroid or Corticoids or Corticoid or Adrenal Cortex Hormone or Cortex Hormone, Adrenal or Hormone, Adrenal Cortex or Triamcinolone acetonide or Acetonide, Triamcinolone or Cinonide or Tricort-40 or Tricort 40 or Tricort40 or Kenalog or Kenalog 40 or Azmacort or Kenacort A or Triesence or dexamethasone intravitreal implant or dexamethasone implant or Ozurdex or Methylfluorprednisolone or Hexadecadrol or Decameth or Decaspray or Dexasone or Dexpak or Maxidex or Millicorten or Oradexon or Decaject or Decaject-L A or Decaject L A or Hexadrol or intravitreal fluocinolone acetonide implant or Iluvien or Acetonide, Fluocinolone or Fluortriamcinolone Acetonide or Acetonide, Fluortriamcinolone or Flurosyn or Jellin or Jellisoft or Synalar or Synalar-HP or Synalar HP or Synemol or Synamol or Alvadermo or Capex or Co-Fluocin or Co Fluocin or Cortiespec or Derma-Smooth FS or Derma Smooth FS or Flucinar or Fluocid or Fluodermo or Fluonid or Fluotrex or Flusolgen or Gelidina).af.
3 (macular edema or Edema, Macular or Irvine-Gass Syndrome or Irvine Gass Syndrome or Syndrome, Irvine-Gass or Cystoid Macular Edema, Postoperative or Macular Edema, Cystoid or Edema, Cystoid Macular or Cystoid Macular Dystrophy or Macular Dystrophy, Dominant Cystoid or Central Retinal Edema, Cystoid or Cystoid Macular Edema).af
4 (intravitreal or Injection, Intravitreal or Injections, Intravitreal or Intravitreal Injection).af.
5 1 and 2 and 3 and 4

For Web of Science：

1 (TS=(Vascular Endothelial Growth Factors) OR TS=(VEGFs) OR TS=(Anti-Vascular Endothelial Growth Factors) OR TS=(Anti-VEGFs) OR TS=(pegaptanib sodium) OR TS=(Macugen) OR TS=(Ranibizumab) OR TS=(Lucentis) OR TS=(RhuFab V2) OR TS=(V2, RhuFab) OR TS=(Bevacizumab) OR TS=(Mvasi) OR TS=(Bevacizumab-awwb) OR TS=(Bevacizumab awwb) OR TS=(Avastin) OR TS=(Aflibercept) OR TS=(VEGF Trap - regeneron) OR TS=(VEGF Trap-Eye) OR TS=(VEGF-Trap) OR TS=(eylea) OR TS=(Zaltrap) OR TS=(AVE 0005) OR TS=(AVE0005) OR TS=(AVE-0005) OR TS=(AVE 005) OR TS=(AVE005) OR TS=(AVE-005) OR TS=(ZIV-aflibercept) OR TS=(Brolucizumab) OR TS=(Beovu) OR TS=(RTH258) OR TS=(RTH-258) OR TS=(ESBA1008) OR TS=(ESBA-1008) OR TS=(Susvimo) OR TS=(Genentech) OR TS=(Abicipar pegol) OR TS=(anti-VEGF DARPin) OR TS=(Faricimab) OR TS=(Vabysmo) OR TS=(Tarcocimab) OR TS=(KH902 fusion protein) OR TS=(conbercept))
2 (TS=(Adrenal Cortex Hormones) OR TS=(Hormones, Adrenal Cortex) OR TS=(Corticosteroids) OR TS=(Corticosteroid) OR TS=(Corticoids) OR TS=(Corticoid) OR TS=(Adrenal Cortex Hormone) OR TS=(Cortex Hormone, Adrenal) OR TS=(Hormone, Adrenal Cortex) OR TS=(Triamcinolone acetonide) OR TS=(Acetonide, Triamcinolone) OR TS=(Cinonide) OR TS=(Tricort-40) OR TS=(Tricort 40) OR TS=(Tricort40) OR TS=(Kenalog) OR TS=(Kenalog 40) OR TS=(Azmacort) OR TS=(Kenacort A) OR TS=(Triesence) OR TS=(dexamethasone intravitreal implant) OR TS=(dexamethasone implant) OR TS=(Ozurdex) OR TS=(Methylfluorprednisolone) OR TS=(Hexadecadrol) OR TS=(Decameth) OR TS=(Decaspray) OR TS=(Dexasone) OR TS=(Dexpak) OR TS=(Maxidex) OR TS=(Millicorten) OR TS=(Oradexon) OR TS=(Decaject) OR TS=(Decaject-L.A.) OR TS=(Decaject L.A.) OR TS=(Hexadrol) OR TS=(intravitreal fluocinolone acetonide implant) OR TS=(Iluvien) OR TS=(Acetonide, Fluocinolone) OR TS=(Fluortriamcinolone Acetonide) OR TS=(Acetonide, Fluortriamcinolone) OR TS=(Flurosyn)

OR TS=(Jellin) OR TS=(Jellisoft) OR TS=(Synalar) OR TS=(Synalar-HP) OR TS=(Synalar HP) OR TS=(Synemol) OR TS=(Synamol) OR TS=(Alvadermo) OR TS=(Capex) OR TS=(Co-Fluocin) OR TS=(Co Fluocin) OR TS=(Cortiespec) OR TS=(Derma-Smooth FS) OR TS=(Derma Smooth FS) OR TS=(Flucinar) OR TS=(Fluocid) OR TS=(Fluodermo) OR TS=(Fluonid) OR TS=(Fluotrex) OR TS=(Flusolgen) OR TS=(Gelidina) OR TS=(triamcinolone acetonide (CLS-TA)))

3 (TS=(macular edema) OR TS=(Edema, Macular) OR TS=(Irvine-Gass Syndrome) OR TS=(Irvine Gass Syndrome) OR TS=(Syndrome, Irvine-Gass) OR TS=(Cystoid Macular Edema, Postoperative) OR TS=(Macular Edema, Cystoid) OR TS=(Edema, Cystoid Macular) OR TS=(Cystoid Macular Dystrophy) OR TS=(Macular Dystrophy, Dominant Cystoid) OR TS=(Central Retinal Edema, Cystoid) OR TS=(Cystoid Macular Edema))

4 (TS=(intravitreal) OR TS=(Injection, Intravitreal) OR TS=(Injections, Intravitreal) OR TS=(Intravitreal Injection))

5 #1 AND #2 AND #3 AND #4

For the cochance library：

1. MeSH descriptor: [Vascular Endothelial Growth Factors] in all MeSH products

2. (VEGFs):ti,ab,kw OR (Anti-Vascular Endothelial Growth Factors):ti,ab,kw OR (Anti-VEGFs):ti,ab,kw OR (pegaptanib sodium):ti,ab,kw OR (Macugen):ti,ab,kw OR (Ranibizumab):ti,ab,kw OR (Lucentis):ti,ab,kw OR (RhuFab V2):ti,ab,kw OR (V2, RhuFab):ti,ab,kw OR (Bevacizumab):ti,ab,kw OR (Mvasi):ti,ab,kw OR (Bevacizumab-awwb):ti,ab,kw OR (Bevacizumab awwb):ti,ab,kw OR (Avastin):ti,ab,kw OR (Aflibercept):ti,ab,kw OR (VEGF Trap-regeneron):ti,ab,kw OR (VEGF Trap-Eye):ti,ab,kw OR (VEGF-Trap):ti,ab,kw OR (eylea):ti,ab,kw OR (Zaltrap):ti,ab,kw OR (AVE 0005):ti,ab,kw OR (AVE0005):ti,ab,kw OR (AVE-0005):ti,ab,kw OR (AVE 005):ti,ab,kw OR (AVE005):ti,ab,kw OR (AVE-005):ti,ab,kw OR (ZIV-aflibercept):ti,ab,kw OR (Brolucizumab):ti,ab,kw OR (Beovu):ti,ab,kw OR (RTH258):ti,ab,kw OR (RTH-258):ti,ab,kw OR (ESBA1008):ti,ab,kw OR (ESBA-1008):ti,ab,kw OR (Susvimo):ti,ab,kw OR (Genentech):ti,ab,kw OR (Abicipar pegol):ti,ab,kw OR (anti-VEGF DARPin):ti,ab,kw OR (Faricimab):ti,ab,kw OR (Vabysmo):ti,ab,kw OR (Tarcocimab):ti,ab,kw OR (KH902 fusion protein):ti,ab,kw OR (conbercept):ti,ab,kw

3.#1 OR #2

4. MeSH descriptor: [Adrenal Cortex Hormones] explode all trees

5. (Hormones, Adrenal Cortex):ti,ab,kw OR (Corticosteroids):ti,ab,kw OR (Corticosteroid):ti,ab,kw OR (Corticoids):ti,ab,kw OR (Corticoid):ti,ab,kw OR (Adrenal Cortex Hormone):ti,ab,kw OR (Cortex Hormone, Adrenal):ti,ab,kw OR (Hormone, Adrenal Cortex):ti,ab,kw OR (Triamcinolone acetonide):ti,ab,kw OR (Acetonide, Triamcinolone):ti,ab,kw OR (Cinonide):ti,ab,kw OR (Tricort-40):ti,ab,kw OR (Tricort 40):ti,ab,kw OR (Tricort40):ti,ab,kw OR (Kenalog):ti,ab,kw OR (Kenalog 40):ti,ab,kw OR (Azmacort):ti,ab,kw OR (Kenacort A):ti,ab,kw OR (Triesence):ti,ab,kw OR (dexamethasone intravitreal implant):ti,ab,kw OR (dexamethasone implant):ti,ab,kw OR (Ozurdex):ti,ab,kw OR (Methylfluorprednisolone):ti,ab,kw OR (Hexadecadrol):ti,ab,kw OR (Decameth):ti,ab,kw OR (Decaspray):ti,ab,kw OR (Dexasone):ti,ab,kw OR (Dexpak):ti,ab,kw OR (Maxidex):ti,ab,kw OR (Millicorten):ti,ab,kw OR (Oradexon):ti,ab,kw OR (Decaject):ti,ab,kw OR (Decaject-L.A.):ti,ab,kw OR (Decaject L.A.):ti,ab,kw OR (Hexadrol):ti,ab,kw OR (intravitreal fluocinolone acetonide implant):ti,ab,kw OR (Iluvien):ti,ab,kw OR (Acetonide,

Fluocinolone):ti,ab,kw OR (Fluortriamcinolone Acetonide):ti,ab,kw OR (Acetonide, Fluortriamcinolone):ti,ab,kw OR (Flurosyn):ti,ab,kw OR (Jellin):ti,ab,kw OR (Jellisoft):ti,ab,kw OR (Synalar):ti,ab,kw OR (Synalar-HP):ti,ab,kw OR (Synalar HP):ti,ab,kw OR (Synemol):ti,ab,kw OR (Synamol):ti,ab,kw OR (Alvadermo):ti,ab,kw OR (Capex):ti,ab,kw OR (Co-Fluocin):ti,ab,kw OR (Co Fluocin):ti,ab,kw OR (Cortiespec):ti,ab,kw OR (Derma-Smooth FS):ti,ab,kw OR (Derma Smooth FS):ti,ab,kw OR (Flucinar):ti,ab,kw OR (Fluocid):ti,ab,kw OR (Fluodermo):ti,ab,kw OR (Fluonid):ti,ab,kw OR (Fluotrex):ti,ab,kw OR (Flusolgen):ti,ab,kw OR (Gelidina):ti,ab,kw OR (triamcinolone acetonide (CLS-TA)):ti,ab,kw

6. #4 OR #5

7. MeSH descriptor: [Macular Edema] explode all trees

8. (Edema, Macular):ti,ab,kw OR (Irvine-Gass Syndrome):ti,ab,kw OR (Irvine Gass Syndrome):ti,ab,kw OR (Syndrome, Irvine-Gass):ti,ab,kw OR (Cystoid Macular Edema, Postoperative):ti,ab,kw OR (Macular Edema, Cystoid):ti,ab,kw OR (Edema, Cystoid Macular):ti,ab,kw OR (Cystoid Macular Dystrophy):ti,ab,kw OR (Macular Dystrophy, Dominant Cystoid):ti,ab,kw OR (Central Retinal Edema, Cystoid):ti,ab,kw OR (Cystoid Macular Edema):ti,ab,kw

9. #7 OR #8

10. MeSH descriptor: [Intravitreal Injections] explode all trees

11. (intravitreal):ti,ab,kw OR (Injection, Intravitreal):ti,ab,kw OR (Injections, Intravitreal):ti,ab,kw

12. #10 OR #11

13. #3 AND #6 AND #9 AND #12

Supplement File S2—Supplementary Tables and Figures

Table S1: The summary of abnormal IOP elevation events.

Author; Publication Year	YesC	NoC	YesM	NoM
Petrovic, N. (2023)	6	6	0	12
Shahid, M.H. (2022)	6	14	0	20
Limon, U. (2021)	3	32	0	30
Chiung-Yi, C. (2021)	3	20	0	30
Entezari, M. (2019)	0	24	0	24
Eris, E. (2019)	0	38	0	34
Maturi, R.K. (2018)	19	46	0	64
Maturi, R.K. (2015)	6	15	1	18
Shoeibi, N. (2013)	1	40	0	37

YesC: The number of IOP elevation events in combination therapy group, **NoC**: The number of normal IOP events in combination therapy group, **YesM**: The number of IOP elevation events in monotherapy group, **NoM**: The number of normal IOP events in monotherapy group

Table S2: The summary of cataract progression events in phakic and pseudophakic eyes.

Author; Publication Year	YesC	NoC	YesM	NoM
Shahid, M.H. (2022)	0	20	0	20
Limon, U. (2021)	1	34	0	30
Chiung-Yi, C. (2021)	2	21	0	20
Entezari, M. (2019)	0	24	0	24
Eris, E. (2019)	0	38	0	34
Maturi, R.K. (2018)	3	62	0	64
Maturi, R.K. (2015)	9	12	0	19
Shoeibi, N. (2013)	0	41	0	37

YesC: The number of cataract progression events in combination therapy group, **NoC**: The number of eyes without cataract progression in combination therapy group, **YesM**: The number of cataract progression events in monotherapy group, **NoM**: The number of eyes without cataract progression in monotherapy group

Table S3: The summary of cataract progression events for 3 included trials.

Author; Publication Year	YesC	NoC	YesM	NoM
Chiung-Yi, C. (2021)	2	14	0	12
Maturi, R.K. (2018)	3	36	0	32
Maturi, R.K. (2015)	9	12	0	19

YesC: The number of cataract progression events in combination therapy group, **NoC**: The number of eyes without cataract progression in combination therapy group, **YesM**: The number of cataract progression events in monotherapy group, **NoM**: The number of eyes without cataract progression in monotherapy group

Table S4: Quality assessment of included studies through MINORS.

Author; Publication Year	Q1	Q2	Q3	Q4	Q5	Q6	Q7	Q8	Q9	Q10	Q11	Q12	Score(quality)
Petrovic, N. 2023	2	2	2	2	0	2	2	0	2	2	2	2	20
Limon, U. 2021	2	2	2	2	0	2	2	0	2	2	2	2	20
Chiung-Yi, C. 2021	2	2	2	2	0	2	2	0	2	0	2	2	18
Eris, E. 2019	2	2	2	2	1	2	2	0	2	0	1	1	17

Checklist items: 1. A clearly stated aim; 2. Inclusion of consecutive patients; 3. Prospective collection of data; 4. Endpoints appropriate to the aim of the study; 5. Unbiased assessment of the study endpoint; 6. Follow-up period appropriate to the aim of the study; 7. Loss to follow up less than 5%; 8. Prospective calculation of the study size; 9. An adequate control group; 10. Contemporary groups; 11. Baseline equivalence of groups; 12. Adequate statistical analyses. Each item was scored from 0 to 2; 0 indicating that it was not reported in the article evaluated, 1 indicating that it was reported but inadequately, and 2 indicating that it was reported adequately. The maximum possible score is 24 points. For non-comparative studies, an overall score > 12 = high; 8–12 = intermediate; < 8 = low. For comparative studies, > 18 = high; 12–18 = intermediate; < 12 = low.

Table 5: Results of publication bias test.

***p* Value**	**1-m BCVA**	**2-m BCVA**	**3-m BCVA**	**6-m BCVA**	**9-m BCVA**	**12-m BCVA**
Begg's Test	1	0.308	0.764	0.764	1	0.296
Egger's Test	0.772	0.151	0.73	0.761	0.482	0.188
***p* value**	**1-m CMT**	**2-m CMT**	**3-m CMT**	**6-m CMT**	**9-m CMT**	**12-m CMT**
Begg's Test	0.902	0.734	0.711	0.133	1	1
Egger's Test	0.289	0.750	0.909	0.115	0.589	0.785
***p* Value**	**IOP elevation**	**Cataract progression**				
Begg's Test	0.368	0.296				
Egger's Test	0.196	0.143				
Harbord’s test	0.628	0.159				

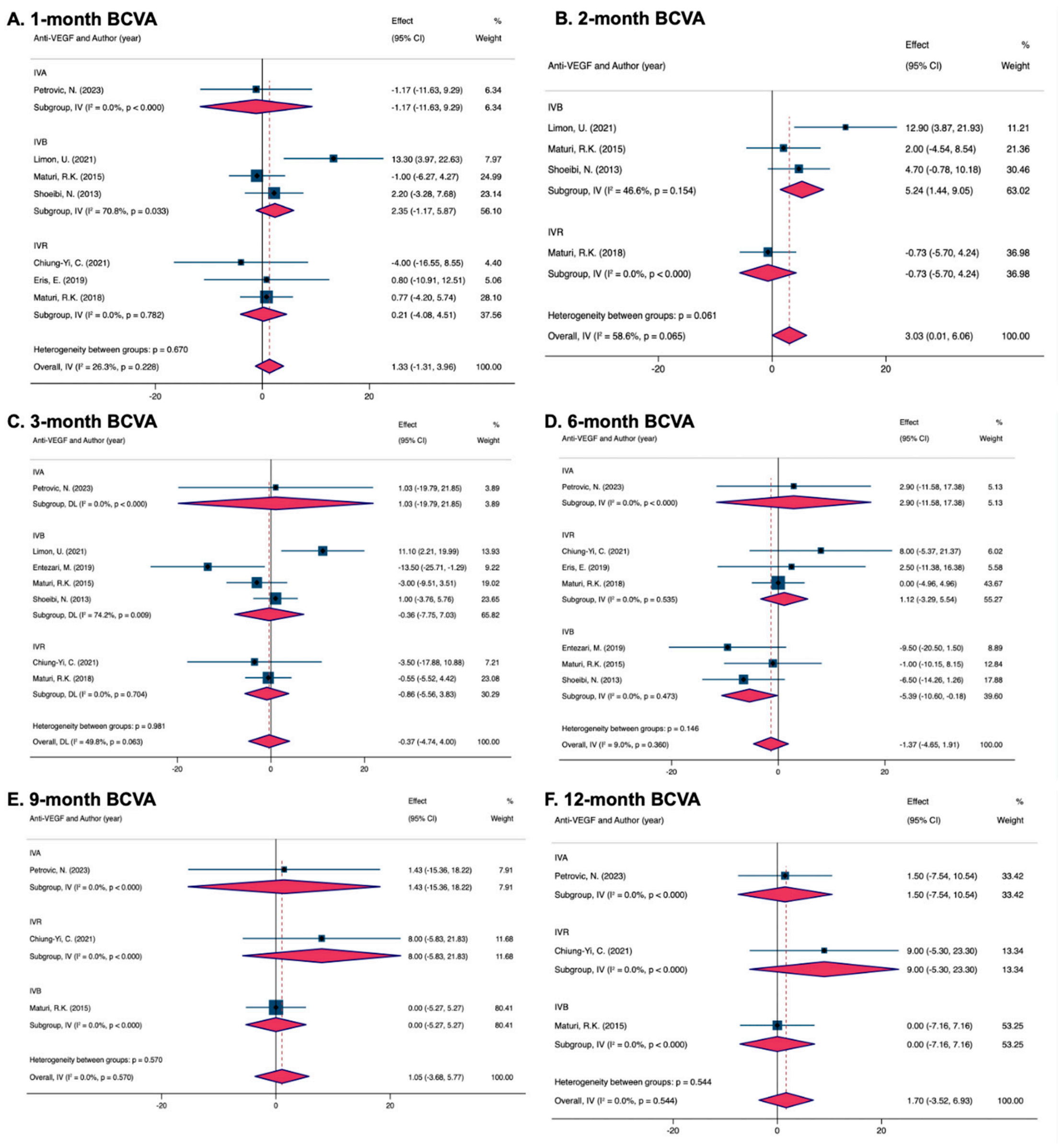

Figure S1: Subgroup analysis for anti-VEGFs. (**A**) for 1-month BCVA; (**B**) for 2-month BCVA; (**C**) for 3-month BCVA; (**D**) for 6-month BCVA; (**E**) for 9-month BCVA; (**F**) for 12-month BCVA; BCVA = best-corrected visual acuity

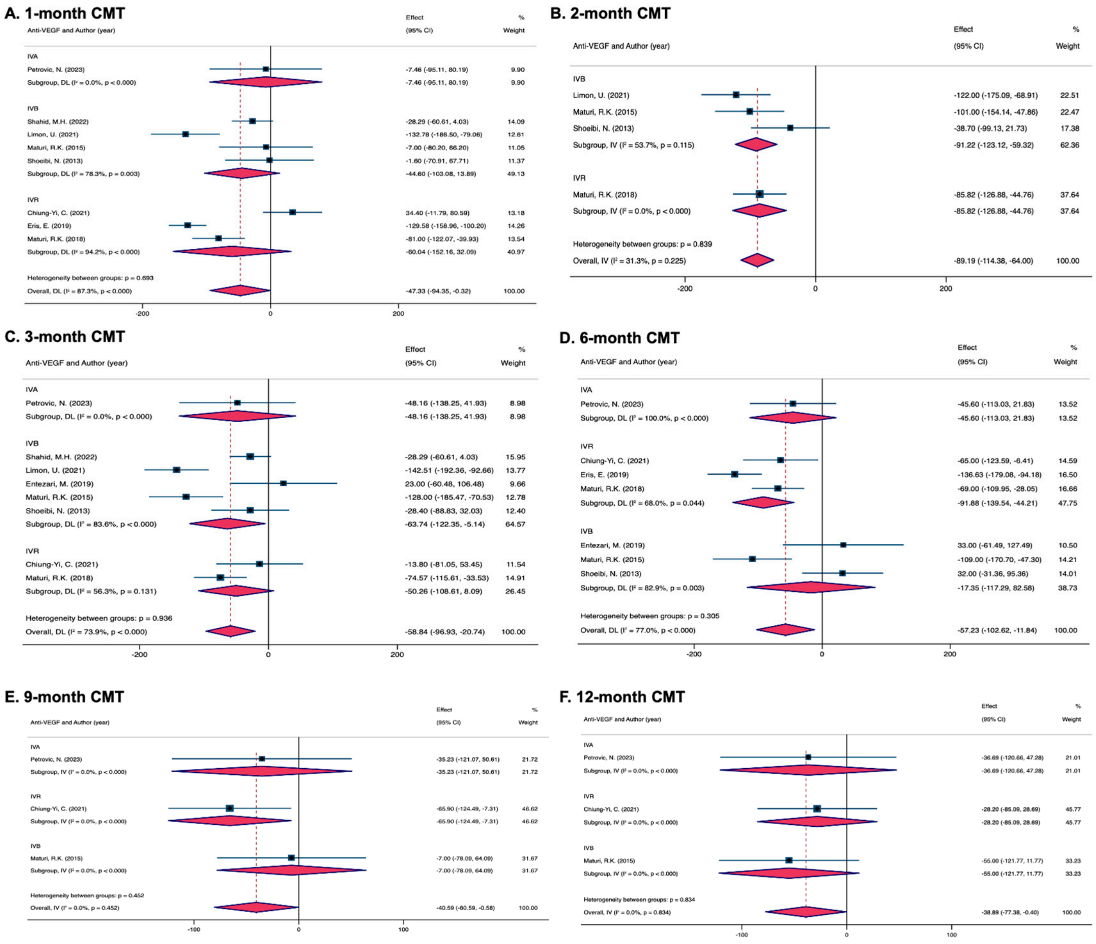

Figure S2: Subgroup analysis for anti-VEGFs. (**A**) for 1-month CMT; (**B**) for 2-month CMT; (**C**) for 3-month CMT; (**D**) for 6-month CMT; (**E**) for 9-month CMT; (**F**) for 12-month CMT; CMT = central retinal thickness

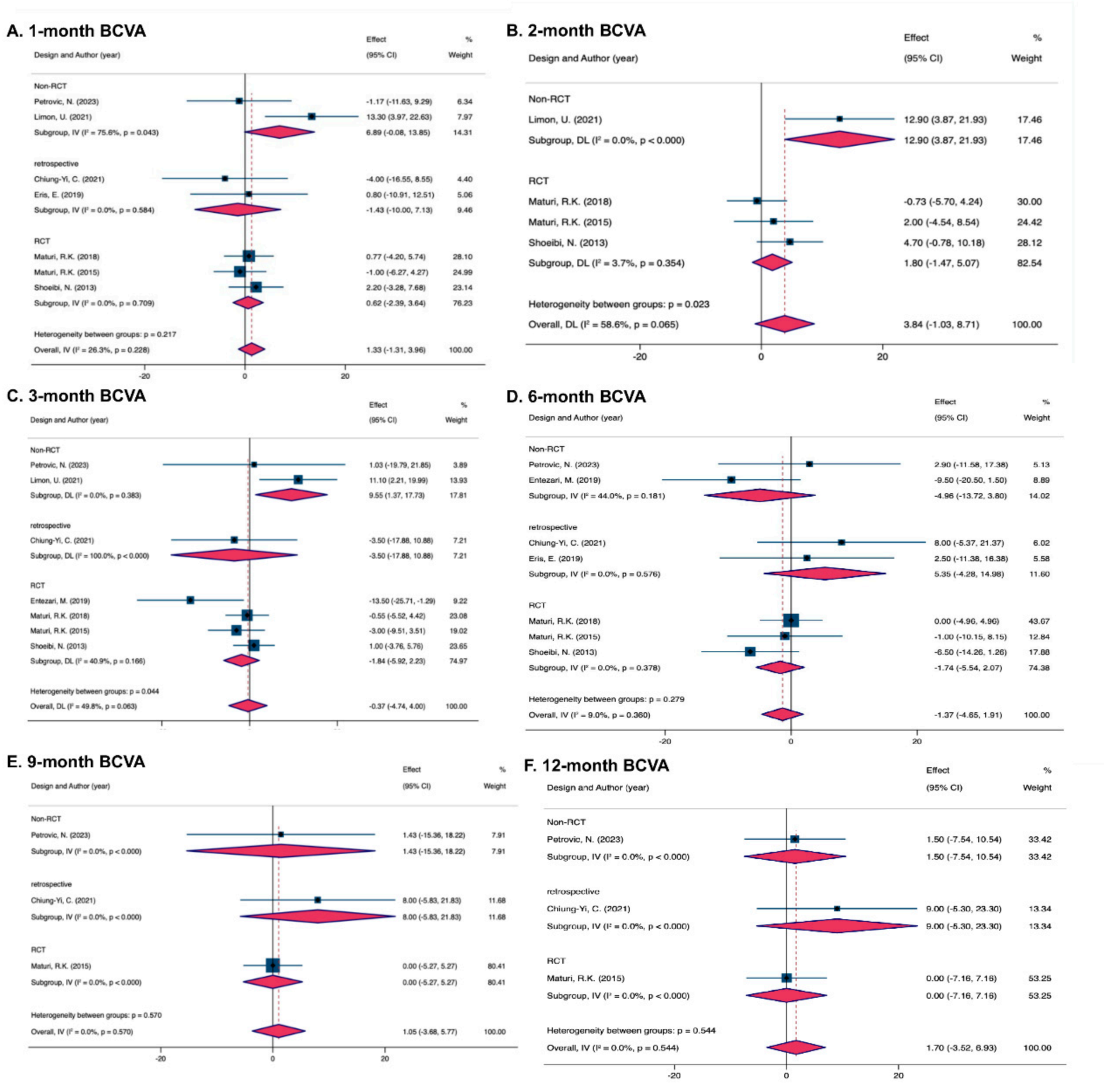

Figure S3: Subgroup analysis for study type. (**A**) for 1-month BCVA; (**B**) for 2-month BCVA; (**C**) for 3-month BCVA; (**D**) for 6-month BCVA; (**E**) for 9-month BCVA; (**F**) for 12-month BCVA; BCVA = best-corrected visual acuity

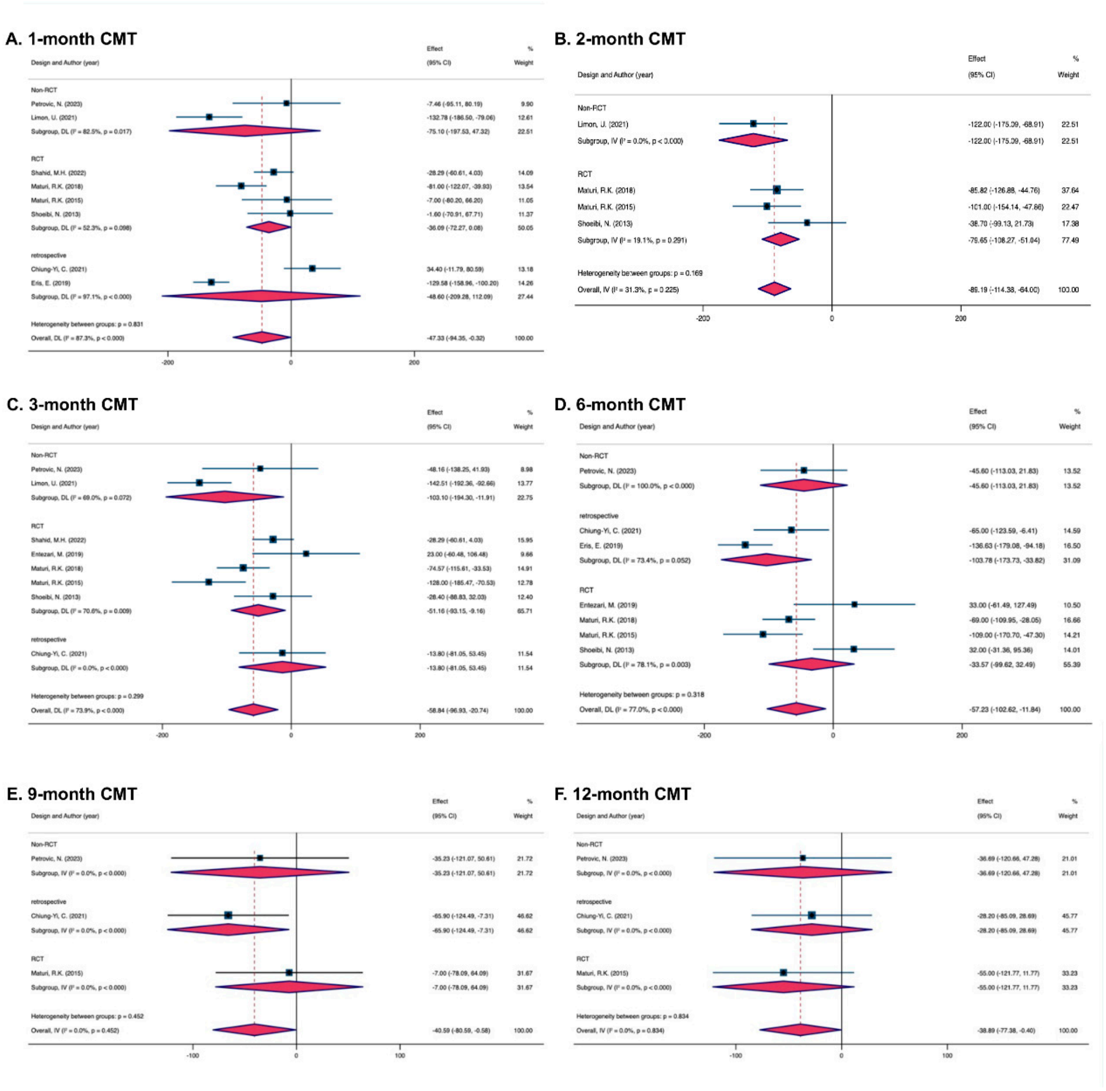

Figure S4: Subgroup analysis for study type. (**A**) for 1-month CMT; (**B**) for 2-month CMT; (**C**) for 3-month CMT; (**D**) for 6-month CMT; (**E**) for 9-month CMT; (**F**) for 12-month CMT; CMT = central retinal thickness

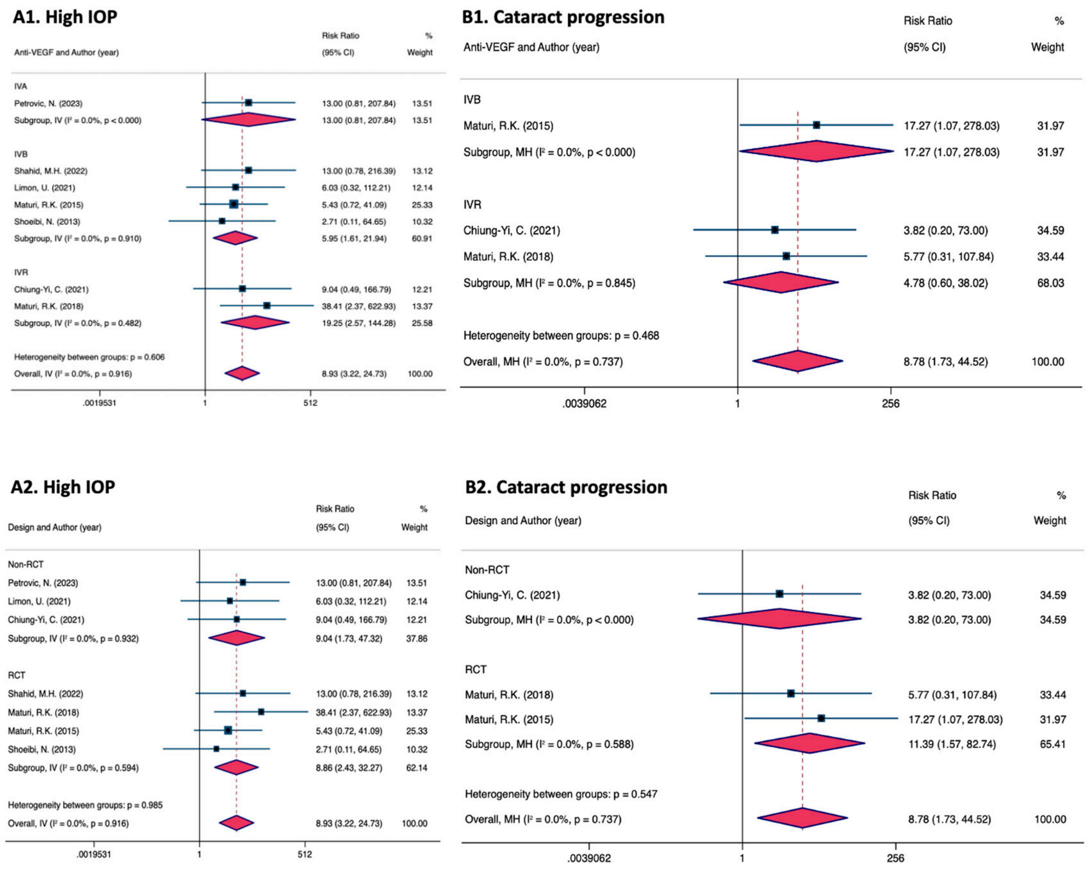

Figure S5: Subgroup analysis for adverse events. (**A1**) High IOP events for different anti-VEGF types; (**B1**) Cataract events for different anti-VEGF; (**A2**) High IOP events for different study designs; (**B2**) Cataract events for different study designs; anti-VEGF = anti-vascular endothelial growth factor; RCT = randomized controlled trials

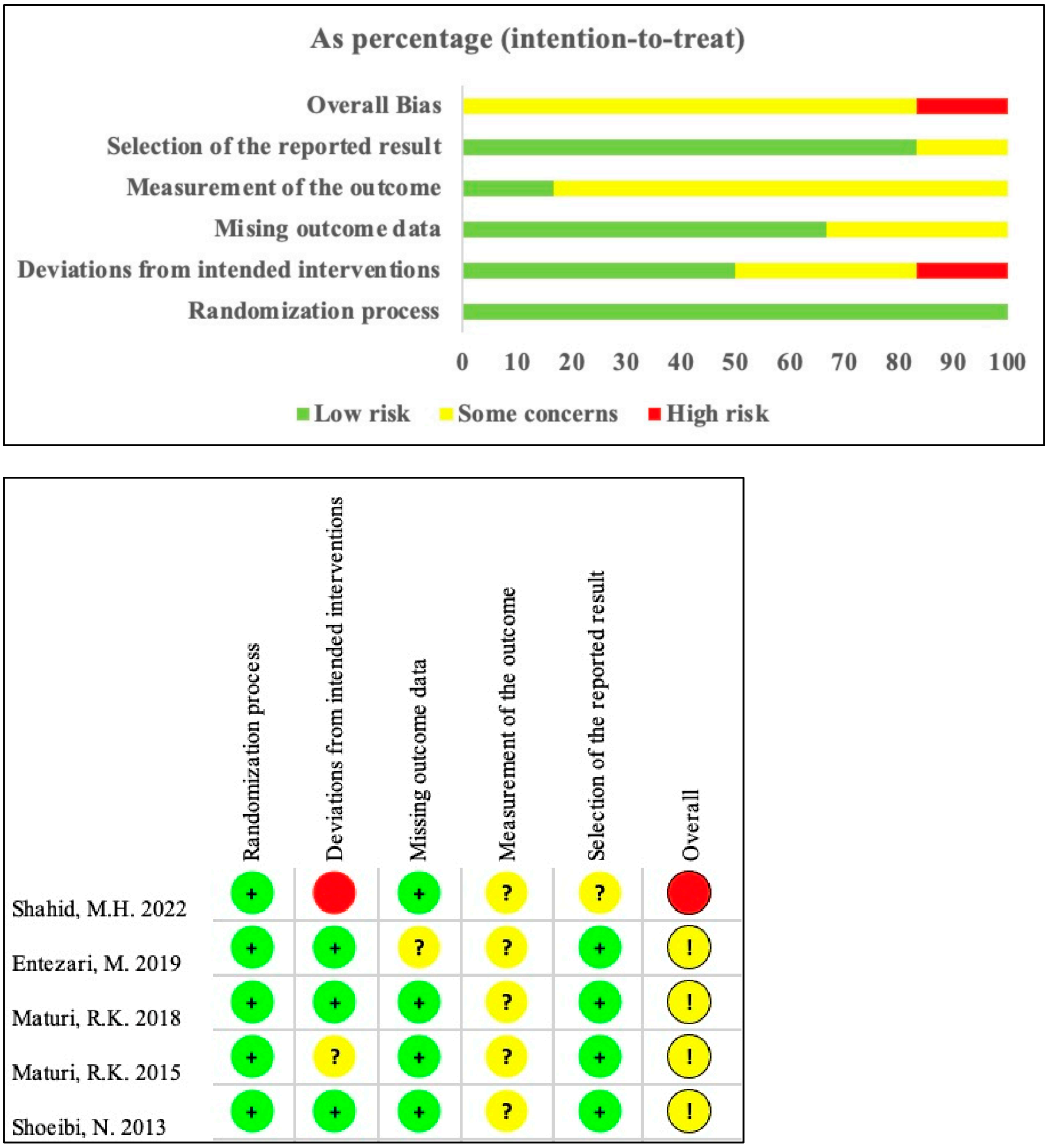

Figure S6: Assessment of the quality of randomized controlled trials according to Cochrane Risk of Bias Tool for Randomized Controlled Trials.

These domains were rated as "high risk of bias", "low risk of bias", or "unclear risk". Finally, an overall risk of bias was determined. The overall risk of bias was "high risk of bias" if at least one domain was deemed "high risk of bias" or if there were "some concerns" in three or more domains. The overall risk of bias was "unclear risk" if there was "unclear risk" in at least one domain. The overall risk of bias was "low risk of bias" if all domains were rated as "low risk of bias".

Other bias: Most studies carry an "unclear risk" in terms of other bias.
